# Salivary cortisol in longitudinal associations between affective symptoms and midlife cognitive function: A British birth cohort study

**DOI:** 10.1016/j.jpsychires.2022.04.007

**Published:** 2022-07

**Authors:** Amber John, Roopal Desai, Rob Saunders, Joshua E.J. Buckman, Barbara Brown, Shirley Nurock, Stewart Michael, Paul Ware, Natalie L. Marchant, Elisa Aguirre, Miguel Rio, Claudia Cooper, Stephen Pilling, Marcus Richards, Darya Gaysina, Josh Stott

**Affiliations:** a ADAPT Lab, Clinical, Educational and Health Psychology, UCL, London, United Kingdom; b Centre for Outcomes and Research Effectiveness, Research Department of Clinical, Educational and Health Psychology, UCL, London, United Kingdom; c iCope – Camden and Islington Psychological Therapies Services, Camden & Islington NHS Foundation Trust, London, United Kingdom; d Division of Psychiatry, UCL, London, United Kingdom; e North East London NHS Foundation Trust (NELFT), London, United Kingdom; f Department of Electronic and Electrical Engineering, UCL, London, United Kingdom; g Camden & Islington NHS Foundation Trust, St Pancras Hospital, London, United Kingdom; h MRC Unit for Lifelong Health and Ageing at UCL, London, United Kingdom; i EDGE Lab, School of Psychology, University of Sussex, Falmer, United Kingdom

**Keywords:** Common mental health problems, Depression, Anxiety, Cognitive function, Cortisol, Longitudinal analysis

## Abstract

Affective disorders are associated with accelerated cognitive ageing. However, current understanding of biological mechanisms which underlie these observed associations is limited. The aim of this study was to test: 1) Whether cortisol acts as a pathway in the association between depressive or anxiety symptoms across adulthood and midlife cognitive function; 2) Whether cortisol is associated with later depressive or anxiety symptoms, and cognitive function. Data were used from the National Child Development Study (NCDS), a sample of infants born in mainland UK during one week of 1958. A measure of the accumulation of affective symptoms was derived from data collected from age 23 to 42 using the Malaise Inventory Scale. Salivary cortisol measures were available at age 44–45. Cognitive function (memory, fluency, information processing) and affective symptoms were assessed at the age of 50. Path models were run to test whether salivary cortisol explained the longitudinal association between depressive or anxiety disorder symptoms and cognitive function. Direct effects of affective symptoms are shown across early to middle adulthood on cognitive function in midlife (memory and information processing errors). However, there were no effects of affective symptoms on cognitive function through cortisol measures. Additionally, cortisol measures were not significantly associated with subsequent affective symptoms or cognitive function at the age of 50. These results do not provide clear evidence to suggest that cortisol plays a role in the association between affective symptoms and cognitive function over this period of time. These findings contribute to our understanding of how the association between affective symptoms and cognitive function operates over time.

## Highlights

• Biological mechanisms underlying links between depression and cognition are unclear.
• The aim of this study was to test the role of cortisol in this association.
• These results provide no evidence that cortisol mediates this association.
• Findings contribute to understanding of how this association operates over time.

## 1 Introduction

Affective disorders (including depression and anxiety) are associated with accelerated cognitive ageing, including faster cognitive decline (John et al., 2018) and increased risk of dementia (Da Silva et al., 2013; Gimson et al., 2018; Gulpers et al., 2016). Associations between affective symptoms and cognitive function can be observed as early as in midlife (age 50) (John et al., 2019). Additionally, according to complementary evidence from two different birth cohort studies based in Britain, accumulation of affective symptoms across adulthood is a more important predictor of cognitive outcomes in midlife (John et al., 2019) and in early old age (James et al., 2018) than affective symptoms present at particular ages (sensitive periods). In the context of a rapidly ageing population, increasing understanding of the mechanistic links between affective symptoms and cognitive ageing may offer insight into how to maintain cognitive health for longer.

One of the proposed potential pathways for the association between affective symptoms and cognitive function is cortisol production and associated hippocampal atrophy. Specifically, altered hypothalamic-pituitary-adrenal (HPA) activity and cortisol hypersecretion are common in patients with major depressive disorder (MDD), and increased cortisol secretion can be observed in approximately 50% of patients with depression (Checkley, 1996). There may also be prolonged abnormal HPA activity which persists after recovery from depression (Bhagwagar et al., 2003; Zobel et al., 2001), and this can be observed even after 20 years from the last depressive episode (Beluche et al., 2009). Changes in cortisol patterns have also been observed in people with anxiety disorders (Hek et al., 2013). Cortisol can be measured by looking at overall levels and at daily fluctuations. Specifically, cortisol has a distinct circadian rhythm, meaning that it is important to test fluctuations in levels across the day.

A recent literature review reported that there is growing evidence for an association between increased cortisol and late-life cognitive function, cognitive decline and dementia (Ouanes and Popp, 2019). One study used data from community based longitudinal cohort studies (the National Survey of Health and Development and Whitehall II) to test associations between cortisol diurnal variation and cognitive functioning (Tsui et al., 2020). This study showed that there was a longitudinal association between increased AM:PM cortisol ratio (diurnal variation) and better cognitive function in later life. The evidence to date has shown that glucocorticoids are associated with key hallmarks of the pathogenesis of dementia, including amyloid beta formation, tau accumulation (Green et al., 2006), and tau hyperphosphorylation (Yang et al., 2014). Persistently elevated glucocorticoids may also contribute to the pathogenesis of dementia by damaging brain structure and function, particularly the hippocampus (Lupien et al., 1998), by promoting oxidative stress, or by contributing to metabolic syndrome and neuroinflammation (Butters et al., 2008; Ouanes and Popp, 2019). More detailed information on the association between cortisol and dementia has been published on elsewhere (Ouanes and Popp, 2019; Zheng et al., 2020).

It is important to look at cognitive function in midlife as an outcome, because dementia has a long preclinical period of several decades. Testing associations exclusively in older adults may therefore produce biased or misleading results, as associations may be due to reverse causality from underlying dementia pathology. Therefore, testing these relationships earlier in the life course before any underlying dementia pathology is likely may be an important way to maximize the likelihood of forward temporal association.

**Fig. 1:**
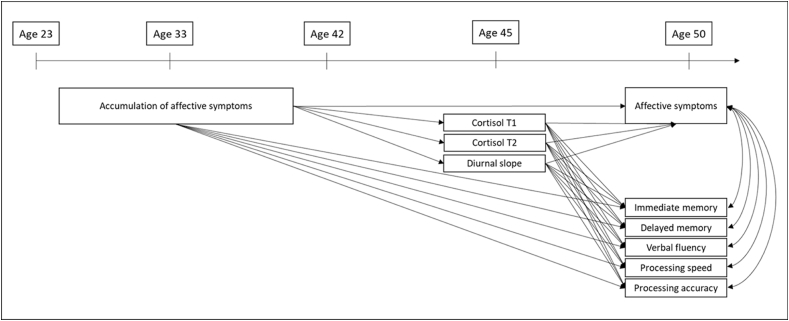
Theoretical model.

However, little research has focused on directly testing cortisol in longitudinal associations between affective symptoms across adulthood and midlife cognitive function. There are at least two ways in which cortisol may contribute to associations between affective symptoms and midlife cognitive function. Firstly, there may be an indirect pathway between affective symptoms and cognitive outcomes which operates through cortisol levels. It is also possible that cortisol may act as a common cause mechanism, whereby cortisol levels may be associated with later affective symptoms and cognitive function, leading to an observed association between the two. These possibilities are not mutually exclusive. Therefore, the aims of this study were to use longitudinal data (between ages 23 and 50) from a population-based birth cohort to test these two processes (see Fig. 1). The hypotheses of this study are that (1) there will be an indirect pathway between affective symptoms and cognitive outcomes which operates through cortisol levels, and; (2) cortisol will be associated with later affective symptoms and cognitive function.

## 2 Material and methods

### 2.1 Participants

Data were used from the National Child Development Study (NCDS; 1958 British Birth Cohort). The sample is comprised of 17,638 men and women born in mainland UK during one week of 1958. Cohort members provided data at regular intervals from birth through to age 55 (the latest available data). Biomedical data were collected from a sub-sample of cohort members (N = 9377) at age 44–45. Previous research has shown that the participating sample at age 44–45 is broadly representative of the full cohort who survived up to this age (Atherton et al., 2008). Written informed consent was provided by all cohort members and ethical approval for the biomedical sweep at age 44/45 was obtained from the South East Multicentre Research Ethics Committee. Further ethical approval for this analysis has been provided by the University of Sussex (ER/AJ316/2). The investigation was carried out in accordance with the latest version of the Declaration of Helsinki.

### 2.2 Measures

#### 2.2.1 Adult cognitive function

Five measures of cognitive function were collected at age 50 in the NCDS: Immediate memory, delayed memory, verbal fluency, information processing speed, and information processing errors. Memory was captured using a 10-item word recall task, with an immediate and a delayed component. Verbal fluency was measured using the Animal Naming Task, in which participants named as many animals as possible within 60s. A letter cancellation task was used to measure information processing speed and processing errors. Processing speed was represented by the number of letters scanned, and processing errors was represented by the number of errors made. For memory, verbal fluency and processing speed, higher scores are indicative of better performance. Conversely, higher scores on the processing errors measure represents poorer performance.

#### 2.2.2 Affective symptoms

Measures of affective symptoms were available at ages 23, 33, 42 and 50. Symptoms were captured with the Malaise Inventory Scale, a measure designed to test for psychological distress and related somatic symptoms (Rodgers et al., 1999). For the purposes of this study, the Malaise Inventory Scale was used as a measure of affective symptoms. Previous research has reported that this scale has acceptable internal consistency (Rodgers et al., 1999), reasonable stability over time (McGee et al., 1986), and good psychometric properties (McGee et al., 1986). The specific questions included in the measure can be accessed in previous publications (Rodgers et al., 1999). At ages 23, 33 and 42 a 24-item version of the Malaise Inventory Scale was administered. The continuous scores were dichotomized using a validated threshold (≥8 out of 24). The binary measures of affective symptoms from age 23 to 42 were summed to derive an overall measure of accumulation prior to cortisol measurement (at age 44–45). The derived accumulation score ranged from 0 to 3 and represented the count of how many time points in which cohort members reported a score above the threshold on the Malaise Inventory Scale. This method has been employed in previous research to capture accumulation of affective symptoms over the life course (John et al., 2020b). At age 50, a shorter 9-item version of the Malaise Inventory Scale was administered (Rodgers et al., 1999).

#### 2.2.3 Cortisol

Measures of salivary cortisol were available at age 44–45. Participants were instructed to collect two saliva samples on the same day. The first sample was collected 45 min after waking up, and the second sample was collected 3h later. Cohort members were asked to refrain from eating, drinking, or brushing their teeth in the 15 min prior to collecting each sample. In order to collect the saliva samples, cohort members chewed on a salivette until it was soaked, and the date and time was recorded for each. The samples were stored at room temperature and mailed to the University of Dresden, where cortisol was measured using a commercial immunoassay kit with chemiluminescence detection (CLIA, IBL-Hamburg, Germany). Detailed information about saliva sample analysis is published and available online (Fuller et al., 2006).

Cortisol data were cleaned in line with previously reported procedures (Stafford et al., 2017). Outlying cortisol values (defined as >100 nmol/L) were excluded, to avoid extreme values exerting a disproportionate effect on results. In addition, cohort members who reported taking endocrine system medications were also excluded, due to known effects of these medications on cortisol profiles. Morning cortisol samples that were not collected between 5:00 and 12:00 were excluded, because individuals with atypical or irregular sleeping patterns may show markedly different cortisol profiles (Stafford et al., 2017). Cortisol has a distinct circadian rhythm, meaning the time of day of sampling has a marked effect on cortisol levels. Specifically, cortisol production builds up overnight to a peak in early morning, and then gradually declines through the day. To account for the time of sampling in this analysis, a linear regression was fit to model associations between cortisol and time of measurement. The residuals of the model were then added to the overall mean cortisol value. A measure of morning variation in cortisol was also derived by subtracting the early morning cortisol value from the late morning value and then dividing by the time between sample collections. In line with previously published procedures using these data, early morning and late morning cortisol values were logₑ transformed, due to positive skew. In addition, participants were excluded who reported completing the second sample before the first sample, or if valid data were not available for one of these time points. Therefore, in total there are three measures of cortisol, used for the analyses: cortisol T1, cortisol T2, and morning variation.

#### 2.2.4 Covariates

Covariates selected for this analysis were sex, childhood cognitive function, childhood socioeconomic position, and education. Childhood cognitive function was measured using a general ability test which cohort members completed at school at age 11 (Range: 0–80). This measure approximated an IQ test and was comprised of a verbal (Range: 0–14) and a non-verbal (Range: 0–40) section. A measure of childhood socioeconomic position at age 11 was derived based on parental occupation and household tenure and was divided into three groups: working, intermediate, and middle. These groups were chosen and derived based on guidelines published by the Centre for Longitudinal Studies (CLS) (Elliott and Lawrence, 2014). Education was measured using the highest level of education achieved by age 50 and was divided into three groups: no education, GCSE to A-Level (or Scottish equivalent), higher education.

### 2.3 Analysis plan

Missing data were explored by comparing the sample with complete information on all key measures and covariates with the sample with missing data. Due to significant differences observed between the sample with complete information and those with missing data (see Results), full information maximum likelihood (FIML) was used to account for missingness (Enders and Bandalos, 2001).

A path model was constructed to test cortisol as an indirect pathway in the association between affective symptoms across adulthood and midlife cognitive function. Specifically, the model included: 1. direct associations between accumulation of affective symptoms from age 23 to 42 and each of the five cognitive outcomes at age 50; 2. indirect associations operating through the salivary cortisol measures at age 44–45. The model also included direct associations between the three cortisol measures available at age 44–45 with cognitive function and affective symptoms at age 50 to test the hypothesis that salivary cortisol may be associated with later affective symptoms, rather than in the opposite direction. The theoretical model is presented in Fig. 1.

Model fit was assessed using standard fit indices: Chi square goodness of fit, Comparative Fit Index (CFI), Tucker Lewis Index (TLI), and Root Mean Square Error of Approximation (RMSEA). Non-significant covariances were removed from the model to improve fit (processing speed with delayed memory; processing errors with immediate memory; processing errors with verbal fluency). Initial models were run unadjusted and then subsequent models were adjusted for all covariates (sex, childhood cognitive function, childhood socioeconomic position, and education). Models were also run using bias corrected bootstrapped 95% confidence intervals (using 1000 bootstraps).

The adjusted model was also run stratified by sex to test whether different patterns of associations existed for men and women. The stratified model did not significantly improve model fit (Stratified model: χ^2^(6) = 10.46. Non-stratified model: χ^2^(3) = 10.26, Chi-square difference: χ^2^(3) = 0.20, *p* > .05). As such, sex was used as a covariate in analyses, rather than as a stratifying variable. All analyses were conducted using RStudio and Mplus.

## 3 Results

### 3.1 Descriptive statistics and missing data analysis

Those with complete information on all variables (N = 3092) achieved significantly higher scores on immediate memory (*p* < .001), delayed memory (*p* < .001), verbal fluency (*p* = .001), and information processing errors (*p* = .003) tests than those with missing data (N = 6285). Those with complete information also had significantly fewer episodes of affective symptoms from age 23 to 42 (*p* < .001), lower affective symptoms at age 50 (*p* < .001), higher childhood cognitive function (*p* < .001), higher socioeconomic position at age 11 (*p* < .001), and higher levels of education by age 50 (*p* < .001) than those with missing data. Those with complete information did not differ from those with missing data on cortisol levels (early morning cortisol sample (log): *p* = .73, late morning cortisol sample (log): *p* = .08, morning variation: *p* = .82), information processing speed scores (*p* = .06), and sex (*p* = .90). The analytical sample available for the study was 6514 (for unadjusted models), and 4973 (for adjusted models). Descriptive statistics for individuals included in fully adjusted models are presented in Table 1. The FIML technique was used to account for missing data and maximize the sample size.

**Table 1:** Demographic information for sample included in unadjusted models (N = 6514).

	N (overall)	N (%) for each level	Mean (SD)
Affective symptoms (age 23 to 42)	6514	–	–
0 times	–	5456 (83.76)	–
1 time	–	724 (11.11)	–
2 times	–	247 (3.79)	–
3 times	–	87 (1.34)	–
Malaise Inventory Scale (above threshold)	–	–	–
Age 23	6514	398 (6.11)	–
Age 33	6514	344 (5.28)	–
Age 42	6514	737 (11.31)	–
Cortisol T1^a^	–	21.24 (10.72)	
Cortisol T2^a^	4317	–	8.2 (6.14)
Morning variation	4243	–	−4.33 (3.71)
Immediate memory	6148	–	6.64 (1.47)
Delayed memory	6103	–	5.53 (1.81)
Verbal fluency	6148	–	22.52 (6.26)
Information processing speed	6043	–	335.2 (88.75)
Information processing errors	6043	–	4.24 (3.88)
Sex	6514	–	–
Male	–	3116 (47.84)	–
Female	–	3398 (52.16)	–
Childhood socioeconomic position	5543	–	–
Middle	–	1221 (22.03)	–
Intermediate	–	2103 (37.94)	–
Working	–	2219 (40.03)	–
Childhood cognitive function	5762	–	46.73 (14.89)
Education	6177	–	–
No academic qualification	–	760 (12.30)	–
GCSE to A Level (or Scottish equivalent)	–	3855 (62.41)	–
Higher education	–	1562 (25.29)	–

a Before log transformation.

### 3.2 Path model

The unadjusted path model was a good fit to the data (N = 6514; χ^2^(3) = 87.10, *p* < .001; CFI = 0.995; TLI = 0.919; RMSEA = 0.066). In the unadjusted model there were significant direct effects of affective symptoms from age 23 to 42 on immediate memory (β = −0.09, SE = 0.01, *p* < .001), delayed memory (β = −0.08, SE = 0.01, *p* < .001), verbal fluency (β = −0.09, SE = 0.01, *p* < .001), and information processing errors (β = 0.05, SE = 0.01, *p* < .001), but not on information processing speed. There were no significant indirect effects through any of the cortisol measures (T1, T2 or morning variation). There were significant total effects of affective symptoms from age 23 to 42 on immediate memory (β = −0.09, SE = 0.01, *p* < .001), delayed memory (β = −0.08, SE = 0.01, *p* < .001), verbal fluency (β = −0.09, SE = 0.01, *p* < .001), and information processing errors (β = 0.05, SE = 0.01, *p* < .001), but not on information processing speed (Table 2). Cortisol at T2 was significantly associated with poorer immediate memory (β = −0.06, SE = 0.02, *p* = .004), delayed memory (β = −0.06, SE = 0.02, *p* = .006), and verbal fluency (β = −0.06, SE = 0.02, *p* = .01) at age 50, but not information processing speed or errors. Cortisol at T1 and morning variation were not significantly associated with any of the subsequent cognitive measures. Additionally, none of the three cortisol measures were significantly associated with subsequent affective symptoms at age 50 (Table 3). The model using bias corrected bootstrapped 95% confidence intervals showed the same pattern of results (Supplementary Table 1).

**Table 2:** Direct, **indirect** and total effects of affective symptoms from age 23 to 42 on cognitive function at age 50.

	Model 1: Unadjusted (N = 6514)	Model 2: Adjusted for Sex (N = 6514)	Model 3: Adjusted for all covariates (N = 4973)
Immediate memory			
Direct effect	**−0.09 (0.01), <.001**^a^	**−0.10 (0.01), <.001**	**−0.05 (0.01), .001**
Indirect effect (through cortisol T1^b^)	0.00 (0.001), .83	−0.001 (0.002), .68	0.001 (0.001), .47
Indirect effect (through cortisol T2)	−0.001 (0.001), .39	−0.001 (0.001), .25	−0.001 (0.001), .36
Indirect effect (through morning variation)	0.00 (0.001), .86	0.001 (0.002), .53	−0.001 (0.002), .49
Total effect	**−0.09 (0.01), <.001**	**−0.10 (0.01), <.001**	**−0.05 (0.01), <.001**
Delayed memory			
Direct effect	**−0.08 (0.01), <.001**	**−0.10 (0.01), <.001**	**−0.05 (0.01), .001**
Indirect effect (through cortisol T1)	−0.002 (0.002), .25	−0.003 (0.002), .17	−0.001 (0.001), .45
Indirect effect (through cortisol T2)	−0.001 (0.001), .39	−0.001 (0.001), .25	−0.001 (0.001), .28
Indirect effect (through morning variation)	0.001 (0.001), .41	0.002 (0.002), .20	0.001 (0.002), .57
Total effect	**−0.08 (0.01), <.001**	**−0.10 (0.01), <.001**	**−0.05 (0.01), <.001**
Verbal fluency			
Direct effect	**−0.09 (0.01), <.001**	**−0.10 (0.01), <.001**	−0.02 (0.01), .19
Indirect effect (through cortisol T1)	−0.001 (0.001), .52	−0.001 (0.002), .49	0.00 (0.001), .82
Indirect effect (through cortisol T2)	−0.001 (0.001), .40	−0.001 (0.001), .26	−0.001 (0.001), .30
Indirect effect (through morning variation)	0.00 (0.001), .71	0.001 (0.002), .61	−0.001 (0.002), .64
Total effect	**−0.09 (0.01), <.001**	**−0.10 (0.01), <.001**	−0.02 (0.01), .15
Processing speed			
Direct effect	0.02 (0.01), .25	−0.002 (0.01), .85	0.02 (0.02), .13
Indirect effect (through cortisol T1)	0.001 (0.001), .64	0.00 (0.002), .80	0.001 (0.001), .67
Indirect effect (through cortisol T2)	0.00 (0.00), .79	0.00 (0.00), .80	0.00 (0.001), .97
Indirect effect (through morning variation)	0.00 (0.001), .87	0.001 (0.002), .73	0.00 (0.002), .97
Total effect	0.02 (0.01), .23	−0.002 (0.01), .90	0.02 (0.02), .12
Processing errors			
Direct effect	**0.05 (0.01), <.001**	**0.05 (0.01), <.001**	**0.04 (0.02), .02**
Indirect effect (through cortisol T1)	0.001 (0.001), .55	0.001 (0.002), .56	0.001 (0.001), .62
Indirect effect (through cortisol T2)	0.00 (0.001), .46	0.001 (0.001), .37	0.00 (0.001), .60
Indirect effect (through morning variation)	−0.001 (0.001), .67	−0.001 (0.002), .69	−0.001 (0.002), .62
Total effect	**0.05 (0.01), <.001**	**0.05 (0.01), <.001**	**0.04 (0.02), .02**

a Presented as: β (SE), *p*.

b Cortisol T1 and T2 values were log transformed.

**Table 3:** Effects of cortisol (T1, T2, and morning variation) on affective symptoms and cognitive function at age 50.

	Model 1: Unadjusted (N = 6514)	Model 2: Adjusted for Sex (N = 6514)	Model 3: Adjusted for all covariates (N = 4973)
Affective symptoms at age 50			
Affective symptoms age 23-42	**0.48 (0.01), <.001**^a^	**0.46 (0.01), <.001**	**0.44 (0.01), <.001**
Cortisol T1	−0.04 (0.03), .22	−0.04 (0.03), .28	−0.07 (0.04), .08
Cortisol T2	0.03 (0.02), .13	0.03 (0.02), .12	0.04 (0.02), .12
Morning variation	−0.05 (0.03), .17	−0.04 (0.03), .30	−0.07 (0.04), .09
Sex	–	**0.10 (0.01), <.001**	**0.09 (0.01), <.001**
Childhood SEP	–	–	−0.004 (0.01), .78
Education	–	–	−0.01 (0.01), .60
Childhood cognition	–	–	**−0.04 (0.01), .008**
Immediate memory			
Affective symptoms age 23-42	**−0.09 (0.01). <.001**	**−0.10 (0.01), <.001**	**−0.05 (0.01), .001**
Cortisol T1	0.01 (0.04), .82	0.02 (0.04), .68	−0.03 (0.04), .40
Cortisol T2	**−0.06 (0.02), .004**	**−0.06 (0.02), .005**	−0.03 (0.02), .23
Morning variation	0.01 (0.04), .86	0.03 (0.04), .52	−0.03 (0.04), .46
Sex	–	**0.12 (0.01), <.001**	**0.08 (0.01), <.001**
Childhood SEP	–	–	−0.02 (0.01), .12
Education	–	–	**0.12 (0.02), <.001**
Childhood cognition	–	–	**0.24 (0.02), <.001**
Delayed memory			
Affective symptoms age 23-42	**−0.08 (0.01), <.001**	**−0.10 (0.01), <.001**	**−0.05 (0.01), <.001**
Cortisol T1	0.05 (0.04), .18	0.06 (0.04), .11	0.03 (0.04), .37
Cortisol T2	**−0.06 (0.02), .006**	**−0.06 (0.02), .006**	−0.04 (0.02), .10
Morning variation	0.04 (0.04), .36	0.06 (0.04), .14	0.02 (0.04), .55
Sex	–	**0.15 (0.01), <.001**	**0.11 (0.01), <.001**
Childhood SEP	–	–	−0.01 (0.01), .41
Education	–	–	**0.12 (0.02), <.001**
Childhood cognition	–	–	**0.26 (0.02), <.001**
Verbal fluency			
Affective symptoms age 23-42	**−0.09 (0.01), <.001**	**−0.10 (0.01), <.001**	−0.02 (0.01), .19
Cortisol T1	0.03 (0.04), .51	0.03 (0.04), .47	−0.01 (0.04), .82
Cortisol T2	**−0.06 (0.02), .01**	**−0.06 (0.02), .01**	−0.04 (0.02), .13
Morning variation	0.01 (0.04), .70	0.02 (0.04), .60	−0.02 (0.04), .63
Sex	–	0.02 (0.01), .11	−0.01 (0.01), .36
Childhood SEP	–	–	**−0.07 (0.01), <.001**
Education	–	–	**0.12 (0.02), <.001**
Childhood cognition	–	–	**0.23 (0.02), <.001**
Processing speed			
Affective symptoms age 23-42	0.02 (0.01), .25	−0.002 (0.01), .85	0.02 (0.02), .13
Cortisol T1	−0.02 (0.04), .63	−0.01 (0.04), .80	−0.02 (0.04), .65
Cortisol T2	−0.01 (0.02), .78	−0.01 (0.02), .80	−0.001 (0.03), .97
Morning variation	−0.01 (0.04), .87	0.01 (0.04), .73	0.002 (0.04), .97
Sex	–	**0.14 (0.01), <.001**	**0.13 (0.01), <.001**
Childhood SEP	–	–	−0.02 (0.02), .18
Education	–	–	**0.08 (0.02), <.001**
Childhood cognition	–	–	**0.06 (0.02), .001**
Processing errors			
Affective symptoms age 23-42	**0.05 (0.01), <.001**	**0.05 (0.01), <.001**	**0.04 (0.02), .02**
Cortisol T1	−0.02 (0.04), .54	−0.02 (0.04), .55	−0.02 (0.04), .60
Cortisol T2	0.03 (0.02), .20	0.03 (0.02), .20	0.01 (0.03), .58
Morning variation	−0.02 (0.04), .67	−0.02 (0.04), .69	−0.02 (0.04), .60
Sex	–	0.02 (0.01), .15	**0.04 (0.01), .009**
Childhood SEP	–	–	−0.02 (0.02), .25
Education	–	–	**0.05 (0.02), .001**
Childhood cognition	–	–	**−0.18 (0.02), <.001**

a Presented as: β (SE), *p*.

The fully adjusted path model was a good fit to the data (N = 4973; χ^2^(3) = 10.26, *p* = .02; CFI = 0.999; TLI = 0.986; RMSEA = 0.022). Findings for the model adjusted for all covariates were similar to those for the unadjusted model. There were significant direct effects of affective symptoms from age 23 to 42 on immediate memory (β = −0.05, SE = 0.01, *p* = .001), delayed memory (β = −0.05, SE = 0.01, *p* < .001), and information processing errors (β = 0.04, SE = 0.02, *p* = .02), but not on verbal fluency or information processing speed. There were no significant indirect effects through any of the three cortisol measures. There were significant total effects of affective symptoms from age 23 to 42 on immediate memory (β = −0.05, SE = 0.01, *p* < .001), delayed memory (β = −0.05, SE = 0.01, *p* < .001), and information processing errors (β = 0.04, SE = 0.02, *p* = .02), but not on verbal fluency or information processing speed (Table 2). In the fully adjusted model, none of the three cortisol measures were significantly associated with any of the subsequent cognitive measures. Additionally, none of the three cortisol measures were significantly associated with subsequent affective symptoms at age 50 (Table 3). The model using bias corrected bootstrapped 95% confidence intervals showed the same pattern of results (Supplementary Table 2).

## 4 Discussion

### 4.1 Summary of findings

The present findings show a clear association between affective symptoms across early to middle adulthood on cognitive function in midlife (immediate memory, delayed memory, and information processing errors), even after adjustment for key covariates. However, no indirect associations between affective symptoms on cognitive function were shown through cortisol. Additionally, cortisol measures were not significantly associated with subsequent affective symptoms or cognitive function at the age of 50. Therefore, our results provide no evidence for cortisol in the association between affective symptoms and cognitive function.

Previous research using these data have provided support for other biomedical pathways in the association between affective symptoms and cognitive outcomes, including inflammation (John et al., 2020b), and cardiometabolic risk (John et al., 2020a). The findings from this study do not provide evidence to support cortisol as one of these pathways in this cohort of people up to age 50 over this length of time.

There are several possible explanations for these findings. Specifically, it is possible that cortisol does not explain the association between affective symptoms and cognitive function, at least up to midlife. Previous research has shown that the extent of hippocampal atrophy in older adults is associated with the duration of time in which hypercortisolemia is experienced (Lupien et al., 1998). In this cohort, the cognitive measures were administered in midlife (age 50), at which age cognitive impairment is a rare outcome. It is possible that the sample were too young for any significant cortisol-related changes to hippocampal function and structure to be present. Hence, as the cohort ages, any potential indirect associations through cortisol may begin to emerge. For example, previous research has found associations between cortisol AM:PM ratio and cognitive function from age 65.9–69.7 (over 15 years later in the life course than in the present study) (Tsui et al., 2020). Another potential explanation for the null finding in this study is that the depressive or anxiety symptoms experienced may not have been sufficiently severe or may not have been experienced for long enough for associations to be observed. Additionally, cortisol is a particularly multi-purpose hormone. These results do not provide evidence for cortisol as an explanatory mechanism of the association between depressive/anxiety symptoms and cognitive function, but they do not necessarily rule out the explanatory value of synchronous physiological processes.

Beyond cortisol, there are many other plausible lifestyle, sociobehavioural and biological mechanisms which may underlie observed associations between affective symptoms and cognitive function. Potential lifestyle and sociobehavioural pathways may include healthy behaviours (e.g. diet, alcohol use, smoking status, etc) and social factors (e.g. social support, isolation, and loneliness). Potential biological pathways may include vascular disease and inflammatory pathways. These processes are not mutually exclusive, and in fact may be synergistic in nature.

### 4.2 Strengths and limitations

These initial findings need to be treated with caution, taking into account the strengths and limitations of the study. Strengths of this study include the use of a large population based cohort. Additionally, repeated measures of affective symptoms were available from young adulthood to midlife. Measures were also captured prospectively from childhood through to midlife, and as such this study avoids limitations associated with recall bias. However, the main limitation of this study is the temporal ordering of the measures available for cortisol, affective symptoms and cognitive function. Specifically, the HPA system is dynamic and cortisol patterns may not be stable across the life course. Therefore, the single assessment of cortisol available in this study may limit interpretability of findings. Additionally, due to the single assessment of cortisol, it was also not possible to determine directionality. Repeated assessment of cortisol would offer more clarity about the direction of associations and would allow more complex modelling of the temporal associations of cortisol with affective symptoms and cognitive function. In addition, there was at least two years between the last available measure of affective symptoms and the collection of cortisol measures. It is therefore not clear whether associations between affective symptoms and cortisol may exist if measures were available contemporaneously.

A further limitation of this study is that measures of cognitive function in adulthood were available at only one time point (age 50), meaning that cognitive trajectories over time could not be modelled. Additionally, cortisol measures were only available 45 min after awakening and 3h later. This means that the full diurnal slope over the course of the day could not be captured. This may affect interpretation of results and as such the findings from this study should be interpreted in this context. Missing data is an inevitable limitation of long-running cohort studies. In this research, missingness was carefully explored and compared with non-missing data. In main analyses, attrition was accounted for using standard statistical techniques (FIML), which produce estimates which are less biased than those produced using complete case analysis (CCA) (Enders and Bandalos, 2001).

### 4.3 Importance

Previous research has shown that late life depression is a potentially modifiable risk factor for dementia (Livingston et al., 2020), and it has been estimated that reducing prevalence of depression by 25% could translate to 827,000 fewer cases of Alzheimer's disease globally (Barnes and Yaffe, 2011). It is important to understand the pathways which link depressive and anxiety symptoms with future cognition, in order for future research to test whether early intervention targeting these pathways can contribute to dementia prevention efforts. The results from this study are not able to provide evidence to support cortisol as a mechanism in this association. These findings are important because they contribute to current understanding of how the association between affective symptoms and cognitive function operates over time.

### 4.4 Conclusion

In conclusion, the results of this study confirm clear associations between affective symptoms on later cognitive function, but provide no evidence to support cortisol as a mechanism within this association, as has been proposed in previous studies and reviews. Future research should focus on further investigating the observed longitudinal link between affective symptoms and cognitive function. Specifically, other potential biomedical and socio-behavioural mechanisms should be tested, in order to further understanding of this association. Future research could also test whether cortisol explains the association between affective symptoms and cognitive outcomes in an older adult sample. Finally, future research should also look at whether individual symptoms of affective symptoms are associated with cortisol and cognitive function.

## Funding

AJ, JS and MR were supported by the Alzheimer's Society (MODIFY Project; Grant number: AS-PG-18-013). 10.13039/100016174AJ and DG were supported by 10.13039/501100000269Economic and Social Research Council (Grant number: ES/J500173/1). 10.13039/100016170JB was supported by the 10.13039/100010269Wellcome Trust through a Clinical Research Fellowship (Grant number: 201292/Z/16/Z). SP, 10.13039/100010309CC and 10.13039/100000092NLM were supported by 10.13039/501100008721UCLH 10.13039/501100000272NIHR 10.13039/100014461Biomedical Research Centre.

## Role of the funding source

The funders had no involvement in study design; in the collection, analysis and interpretation of data; in the writing of the report; and in the decision to submit the article for publication.

## 5 Data statement

Data is available upon application to METADAC.

## 6 Contributors

Amber John was involved in the design of study, running statistical analysis, interpreting results, drafting the article and editing it. Barbara Brown, Shirley Nurock, Stewart Michael, and Paul Ware were involved in critically evaluating the article for important intellectual content and in providing the lay audience perspective on the work. Roopal Desai, Rob Saunders, Joshua E J Buckman, Natalie L Marchant, Elisa Aguirre, Miguel Rio, Claudia Cooper, and Steve Pilling were involved in the interpretation of findings, and critically evaluating the article for important intellectual content. Marcus Richards, Darya Gaysina, and Joshua Stott were involved in the design of the study, the interpretation of data, and critically evaluating the article for important intellectual content.

## CRediT authorship contribution statement

**Amber John:** Conceptualization, Methodology, Software, Formal analysis, Writing – original draft, Writing – review & editing, Project administration. **Roopal Desai:** Writing – review & editing. **Rob Saunders:** Writing – review & editing. **Joshua E.J. Buckman:** Writing – review & editing. **Barbara Brown:** Writing – review & editing. **Shirley Nurock:** Writing – review & editing. **Stewart Michael:** Writing – review & editing. **Paul Ware:** Writing – review & editing. **Natalie L. Marchant:** Writing – review & editing. **Elisa Aguirre:** Writing – review & editing. **Miguel Rio:** Writing – review & editing. **Claudia Cooper:** Writing – review & editing. **Stephen Pilling:** Writing – review & editing. **Marcus Richards:** Conceptualization, Methodology, Writing – review & editing. **Darya Gaysina:** Conceptualization, Methodology, Writing – review & editing, Supervision. **Josh Stott:** Conceptualization, Methodology, Writing – review & editing, Supervision.

## Declaration of competing interest

None.
