# Supplementary material for: Salivary cortisol in longitudinal associations between affective symptoms and midlife cognitive function: A British birth cohort study
Source: J Psychiatr Res. 2022 Jul;151:217–24. doi: 10.1016/j.jpsychires.2022.04.007 (PMC10442295; doi:10.1016/j.jpsychires.2022.04.007)
Supplement: Multimedia component 1 [file mmc1.docx]

**Supplementary Materials**

**Supplementary Table 1:** Table 2: Direct, indirect and total effects of depression and anxiety symptoms from age 23 to 42 on cognitive function at age 50. Model using bias corrected bootstrapped 95% confidence intervals.

**Supplementary Table 2**: Effects of cortisol (T1, T2, and morning variation) on depression and anxiety symptoms and cognitive function at age 50. Model using bias corrected bootstrapped 95% confidence intervals.

**Supplementary Table 1:** Table 2: Direct, indirect and total effects of depression and anxiety symptoms from age 23 to 42 on cognitive function at age 50. Model using bias corrected bootstrapped 95% confidence intervals.

|  | **Model 1: Unadjusted   (N=6514)** | **Model 2: Adjusted for Sex   (N=6514)** | **Model 3: Adjusted for all covariates (N=4973)** |
| --- | --- | --- | --- |
| Immediate memory |  |  |  |
| Direct effect | **-0.09 (-0.12, -0.06), <.001*** | **-0.10 (-0.13, -0.07), <.001** | **-0.05 (-0.08, -0.02), .001** |
| Indirect effect (through cortisol T1) | 0.00 (-0.01, 0.002), .85 | -0.001 (-0.01, 0.002), .71 | 0.001 (-0.001, 0.01), .52 |
| Indirect effect (through cortisol T2) | -0.001 (-0.004, 0.001), .45 | -0.001 (-0.004, 0.001), .30 | -0.001 (-0.004, 0.00), .44 |
| Indirect effect (through morning variation) | 0.00 (-0.002, 0.004), .88 | 0.001 (-0.001, 0.01), .55 | -0.001 (-0.01, 0.001), .51 |
| Total effect | **-0.09 (-0.12, -0.06), <.001** | **-0.10 (-0.13, -0.08), <.001** | **-0.05 (-0.08, -0.02), .001** |
| Delayed memory |  |  |  |
| Direct effect | **-0.08 (-0.11, -0.05), <.001** | **-0.10 (-0.13, -0.07), <.001** | **-0.05 (-0.08, -0.02), .001** |
| Indirect effect (through cortisol T1) | -0.002 (-0.01, 0.00), .26 | -0.003 (-0.01, 0.00), .17 | -0.001 (-0.01, 0.001), .51 |
| Indirect effect (through cortisol T2) | -0.001 (-0.004, 0.001), .44 | -0.001 (-0.004, 0.001), .28 | -0.001 (-0.01, 0.00), .34 |
| Indirect effect (through morning variation) | 0.001 (-0.001, 0.01), .42 | 0.002 (0.00, 0.01), .20 | 0.001 (-0.001, 0.01), .59 |
| Total effect | **-0.08 (-0.11, -0.06), <.001** | **-0.10 (-0.13, -0.08), <.001** | **-0.05 (-0.08, -0.02), .001** |
| Verbal fluency |  |  |  |
| Direct effect | **-0.09 (-0.12, -0.07), <.001** | **-0.10 (-0.12, -0.07), <.001** | -0.02 (-0.05, 0.01), .19 |
| Indirect effect (through cortisol T1) | -0.001 (-0.01, 0.001), .54 | -0.001 (-0.01, 0.001), .49 | 0.00 (-0.001, 0.003), .84 |
| Indirect effect (through cortisol T2) | -0.001 (-0.004, 0.001), .44 | -0.001 (-0.004, 0.00), .30 | -0.001 (-0.004, 0.00), .33 |
| Indirect effect (through morning variation) | 0.00 (-0.002, 0.004), .72 | 0.001 (-0.002, 0.01), .61 | -0.001 (-0.01, 0.001), .62 |
| Total effect | **-0.09 (-0.12, -0.07), <.001** | **-0.10 (-0.12, -0.07), <.001** | -0.02 (-0.05, 0.01), .16 |
| Processing speed |  |  |  |
| Direct effect | 0.02 (-0.01, 0.04), .27 | -0.002 (-0.03, 0.02), .86 | 0.02 (-0.01, 0.06), .15 |
| Indirect effect (through cortisol T1) | 0.001 (-0.002, 0.01), .66 | 0.00 (-0.003, 0.004), .80 | 0.001 (-0.001, 0.01), .73 |
| Indirect effect (through cortisol T2) | 0.00 (-0.002, 0.00), .85 | 0.00 (-0.002, 0.001), .83 | 0.00 (-0.002, 0.001), .97 |
| Indirect effect (through morning variation) | 0.00 (-0.004, 0.003), .88 | 0.001 (-0.002, 0.01), .75 | 0.00 (-0.003, 0.01), .97 |
| Total effect | 0.02 (-0.01, 0.04), .26 | -0.002 (-0.03, 0.03), .91 | 0.02 (-0.01, 0.05), .14 |
| Processing accuracy |  |  |  |
| Direct effect | **0.05 (0.02, 0.08), <.001** | **0.05 (0.02, 0.08), .001** | **0.04 (0.004, 0.07), .02** |
| Indirect effect (through cortisol T1) | 0.001 (-0.002, 0.01), .59 | 0.001 (-0.002, 0.01), .58 | 0.001 (-0.001, 0.01), .71 |
| Indirect effect (through cortisol T2) | 0.00 (0.00, 0.003), .54 | 0.001 (0.00, 0.003), .43 | 0.00 (-0.001, 0.003), .66 |
| Indirect effect (through morning variation) | -0.001 (-0.01, 0.001), .70 | -0.001 (-0.01, 0.002), .71 | -0.001 (-0.01, 0.002), .66 |
| Total effect | **0.05 (0.02, 0.08), <.001** | **0.05 (0.02, 0.08), <.001** | **0.04 (0.01, 0.07), .02** |

* Results presented as β (SE), *p.*

**Supplementary Table 2**: Effects of cortisol (T1, T2, and morning variation) on depression and anxiety symptoms and cognitive function at age 50. Model using bias corrected bootstrapped 95% confidence intervals.

|  | **Model 1: Unadjusted  (N=6514)** | **Model 2: Adjusted for Sex (N=6514)** | **Model 3: Adjusted for all covariates (N=4973)** |
| --- | --- | --- | --- |
| Affective symptoms at age 50 |  |  |  |
| Affective symptoms age 23-42 | **0.48 (0.45, 0.51), <.001*** | **0.46 (0.44, 0.49), <.001** | **0.44 (0.41, 0.47), <.001** |
| Cortisol T1 | -0.04 (-0.11, 0.03), .26 | -0.04 (-0.11, 0.03), .32 | -0.07 (-0.15, 0.01), .13 |
| Cortisol T2 | 0.03 (-0.01, 0.07), .15 | 0.03 (-0.01, 0.07), .14 | 0.04 (-0.01, 0.08), .14 |
| Morning variation | -0.05 (-0.11, 0.03), .19 | -0.04 (-0.10, 0.04), .33 | -0.07 (-0.14, 0.02), .12 |
| Immediate memory |  |  |  |
| Affective symptoms age 23-42 | **-0.09 (-0.12, -0.06). <.001** | **-0.10 (-0.13, -0.07), <.001** | **-0.05 (-0.08, -0.02), .001** |
| Cortisol T1 | 0.01 (-0.06, 0.09), .83 | 0.02 (-0.05, 0.09), .68 | -0.03 (-0.11, 0.04), .38 |
| Cortisol T2 | **-0.06 (-0.11, -0.02), .01** | **-0.06 (-0.11, -0.02), .007** | -0.03 (-0.08, 0.02), .24 |
| Morning variation | 0.01 (-0.06, 0.08), .85 | 0.03 (-0.04, 0.10), .49 | -0.03 (-0.11, 0.04), .45 |
| Delayed memory |  |  |  |
| Affective symptoms age 23-42 | **-0.08 (-0.11, -0.05), <.001** | **-0.10 (-0.13, -0.07), <.001** | **-0.05 (-0.08, -0.02), .001** |
| Cortisol T1 | 0.05 (-0.02, 0.12), .14 | 0.06 (-0.003, 0.13), .08 | 0.03 (-0.04, 0.10), .33 |
| Cortisol T2 | **-0.06 (-0.10, -0.02), .004** | **-0.06 (-0.10, -0.02), .004** | -0.04 (-0.08, 0.01), .09 |
| Morning variation | 0.04 (-0.03, 0.10), .31 | 0.06 (-0.01, 0.12), .10 | 0.02 (-0.05, 0.09), .52 |
| Verbal fluency |  |  |  |
| Affective symptoms age 23-42 | **-0.09 (-0.12, -0.07), <.001** | **-0.10 (-0.12, -0.07), <.001** | -0.02 (-0.05, 0.01), .19 |
| Cortisol T1 | 0.03 (-0.04, 0.10), .48 | 0.03 (-0.04, 0.10), .44 | -0.01 (-0.08, 0.06), .80 |
| Cortisol T2 | **-0.06 (-0.10, -0.01), .01** | **-0.06 (-0.10, -0.01), .01** | -0.04 (-0.08, 0.01), .12 |
| Morning variation | 0.01 (-0.06, 0.08), .68 | 0.02 (-0.05, 0.09), .57 | -0.02 (-0.09, 0.04), .57 |
| Processing speed |  |  |  |
| Affective symptoms age 23-42 | 0.02 (-0.01, 0.04), .27 | -0.002 (-0.03, 0.02), .86 | 0.02 (-0.01, 0.06), .15 |
| Cortisol T1 | -0.02 (-0.09. 0.06), .63 | -0.01 (-0.08, 0.06), .79 | -0.02 (-0.10, 0.06), .64 |
| Cortisol T2 | -0.01 (-0.05, 0.03), .77 | -0.01 (-0.05, 0.03), .78 | -0.001 (-0.04, 0.04), .97 |
| Morning variation | -0.01 (-0.08, 0.07), .86 | 0.01 (-0.06, 0.09), .71 | 0.002 (-0.07, 0.08), .96 |
| Processing accuracy |  |  |  |
| Affective symptoms age 23-42 | **0.05 (0.02, 0.08), <.001** | **0.05 (0.02, 0.08), .001** | **0.04 (0.004, 0.07), .02** |
| Cortisol T1 | -0.02 (-0.10, 0.05), .54 | -0.02 (-0.10, 0.06), .55 | -0.02 (-0.11, 0.06), .60 |
| Cortisol T2 | 0.03 (-0.01, 0.07), .17 | 0.03 (-0.01, 0.07), .17 | 0.01 (-0.04, 0.06), .56 |
| Morning variation | -0.02 (-0.09, 0.05), .65 | -0.02 (-0.09, 0.05), .68 | -0.02 (-0.11, 0.05), .59 |

* Results presented as β (SE), *p.*
